# Terahertz emission from gold nanorods irradiated by ultrashort laser pulses of different wavelengths

**DOI:** 10.1038/s41598-019-39604-5

**Published:** 2019-03-01

**Authors:** Keisuke Takano, Motoki Asai, Kosaku Kato, Hideaki Komiyama, Akihisa Yamaguchi, Tomokazu Iyoda, Yuzuru Tadokoro, Makoto Nakajima, Michael I. Bakunov

**Affiliations:** 1 Center for Energy and Environmental Science, Shinshu University, 4-17-1 Wakasato, Nagano, 380-8553 Japan; 2 Institute of Laser Engineering, Osaka University, 2-6 Yamadaoka, Suita, Osaka 565-0871 Japan; 3 JST-ERATO Iyoda Supra-Integrated Material Project, Tokyo Institute of Technology, 4259 Nagatsuda-Cho, Midori-Ku, Yokohama, Kanagawa 226-8503 Japan; 4 University of Nizhny Novgorod, 23 Gagarin Avenue, Nizhny Novgorod, 603950 Russia

## Abstract

Electron photoemission and ponderomotive acceleration by surface enhanced optical fields is considered as a plausible mechanism of terahertz radiation from metallic nanostructures under ultrafast laser excitation. To verify this mechanism, we studied experimentally terahertz emission from an array of gold nanorods illuminated by intense (~10–100 GW/cm^2^) femtosecond pulses of different central wavelengths (600, 720, 800, and 1500 nm). We found for the first time that the order of the dependence of the terahertz fluence on the laser intensity is, unexpectedly, almost the same (~4.5–4.8) for 720, 800, and 1500 nm and somewhat higher (~6.6) for 600 nm. The results are explained by tunneling currents driven by plasmonically enhanced laser field. In particular, the pump-intensity dependence of the terahertz fluence is more consistent with terahertz emission from the sub-cycle bursts of the tunneling current rather than with the ponderomotive mechanism.

## Introduction

Illumination of a metal surface with ultrashort laser pulses can result in the generation of terahertz radiation^1^. The generation efficiency can be enhanced by using metallic nanostructures^2^, such as gratings^3^, percolated films^4–7^, randomly arranged nanoparticles^8^, nanohole and nanoparticle ordered arrays^6,9^, and metasurfaces^10,11^. This is explained by the plasmonic enhancement of the optical fields. Although the efficiency is, so far, lower than with nonlinear optical crystals, nanostructured metal emitters have a potential of broadbandness and high damage resistance to optical radiation. Moreover, measuring terahertz radiation from optically excited metal nanostructures can be used as a tool to study ultrafast photoelectron emission and plasmonic acceleration. Optically driven plasmonic nanostructures are considered as promising ultrafast photocathodes^12^. From the physical point of view, there is a great interest in understanding the mechanisms involved in the generation of terahertz light from metal surfaces.

Two main physical models have been proposed to explain terahertz generation from metal surfaces. Optical rectification, i.e., a second-order nonlinear optical process, is invoked as a mechanism of terahertz generation at relatively low pump intensities (<1 GW/cm^2^)^1,4,6,7,11,13^. For high pump intensities (>1 GW/cm^2^), terahertz generation is commonly explained by the ponderomotive acceleration of photoejected electrons^3,6,9,14^. Since the metal work function is typically several times larger than the photon energy of near-infrared pump lasers, the latter model invokes multiphoton ionization and, therefore, predicts a high-order dependence of the terahertz yield on the optical intensity. The experimental dependences, indeed, demonstrate high-order nonlinearities but typically lower than the theoretical values^3,6,9,14^. Moreover, the experiments show a crossover from a high-order to a second-order nonlinearity at further increase of the optical intensity (>10 GW/cm^2^). As an explanation, the transition between multiphoton to tunneling regimes of electron emission has been invoked^3,6,9^. The optical intensity dependences of the terahertz emission and photoelectron emission show some correlation^9^. However, one might expect a higher-order dependence for the terahertz emission than for the electron emission, while in experiment they are comparable^9^. Thus, the detailed mechanism of the terahertz generation has not been elucidated.

If multiphoton ionization contributes considerably to the electron emission and by that to terahertz generation, one can expect a wavelength dependence of the nonlinear order of the generation process. In order to verify this assumption, we investigate in this paper terahertz generation from an array of Au nanorods illuminated by femtosecond optical pulses of different central wavelengths. To obtain different wavelengths, we used an optical parametric amplifying (OPA) system (TOPAS, Spectra-Physics). The optical pump intensity was varied in a wide range ~1–100 GW/cm^2^.

## Experiment and Results

**Figure 1:**
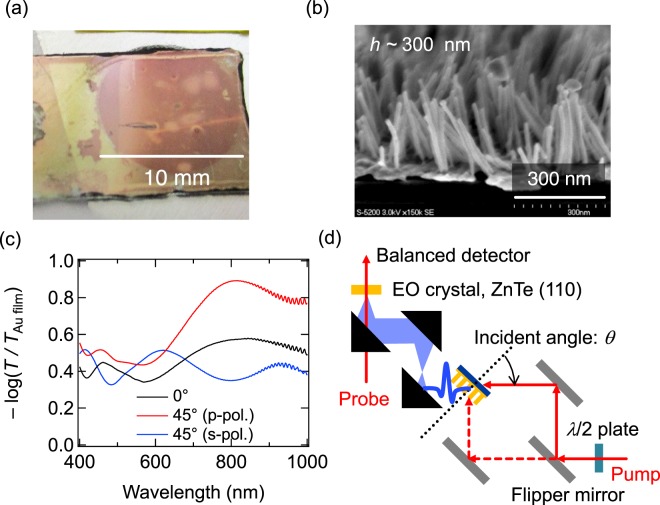
(**a**) Photograph and (**b**) SEM image of the Au nanorod sample. (**c**) Absorbance spectra of the sample for the incident angles of 0° and 45° for *p*- and *s*-polarizations (normalized to the absorbance of the Au-coated glass substrate without nanorods). (**d**) Schematic of the terahertz generation and detection setup.

The Au nanorod sample was fabricated on a Au-coated glass substrate by electroplating with use of a liquid crystalline block copolymer template PEO-*b*-PMA(Az), which is consisted of hydrophilic poly(ethylene oxide) (PEO) and hydrophobic poly(methacrylate) bearing an azobenzene mesogen on the side chain (PMA(Az))^15,16^. The thickness of the Au coating was around 15 nm. The cylindrical nanodomains of the PEO segment in the block copolymer were aligned vertically in a hexagonal array. The period of the array was around 27 nm. The Au nanorods were electroplated through the PEO cylindrical nanodomains to the length of 300 nm and then the copolymer template was removed by reactive ion etching with oxygen. The diameter of the nanorods was around 12 nm. Figure 1(a,b) show the photograph and scanning electron microscope (SEM) image of the Au nanorod sample, respectively. Figure 1(c) shows the absorbance of the Au nanorod sample calculated from the transmission spectra for the normal incidence of light and for the oblique incidence at 45° for *p*- and *s*-polarizations. The absorbance spectra were normalized to those of the Au-coated glass substrate without nanorods. The absorbance shows a broad peak attributed to the longitudinal (with the electron motion along a nanorod) plasmon resonance around 800 nm for the oblique incidence of *p*-polarized light.

We first examine the terahertz generation under the excitation by a Ti:sapphire regenerative amplifier with the central wavelength of 800 nm. The pulse duration and repetition rate were 50 fs and 1 kHz, respectively. The nanorod array was illuminated with *p*-polarized laser radiation from the substrate side or from the air side by switching the optical path with a flipping mirror (Fig. 1(d)). For illumination from the substrate side (transmission geometry), the incident angle *θ* was varying by rotating the sample. For illumination from the air side (reflection geometry), the incident angle was fixed to 45°. For both cases, the generated terahertz pulses were focused on a ZnTe crystal for detection by electro-optic sampling.

**Figure 2:**
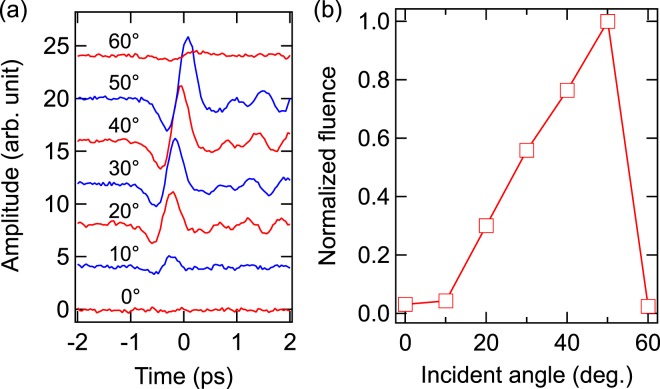
Transmission geometry, excitation at 800 nm. (**a**) Terahertz waveforms for different incident angles. (**b**) Angular dependence of the terahertz pulse energy.

In the transmission geometry, the optical peak intensity was fixed to about 110 GW/cm^2^ and the influence of the incident angle *θ* on the terahertz generation was investigated. Figure 2(a) shows the measured terahertz time-domain waveforms *E*(*t*) for different incident angles in the transmission geometry. Figure 2(b) shows the terahertz pulse energy (the integral of *E*^2^(*t*) over the full pulse duration) as a function of *θ*. There is practically no terahertz emission for normal incidence. Terahertz emission increases with *θ*, exhibits a peak around 50°, and then drops rapidly. Polyushkin *et al*.^6^ observed a similar angular dependence for terahertz emission from arrays of triangular silver nanoparticles. They qualitatively explained the observed dependence by a competition of two main factors, i.e., an increase of the out-of-plane ponderomotive current with *θ* and a simultaneous decrease of the optical intensity due to an increase of the illuminated area. In the transmission geometry of our experiment, angular dependent transmission of the optical pulse through the glass substrate could play a role. However, due to the Brewster effect at the air/glass (with the Brewster angle of ~56°) and glass/air interfaces the power transmission coefficient of the glass substrate for the *p*-polarized laser radiation is close to unity in a wide interval of the incident angles, 0 < *θ* < 75°. Thus, the factor of transmission does not affect noticeably the position of the peak in Fig. 2(b).

**Figure 3:**
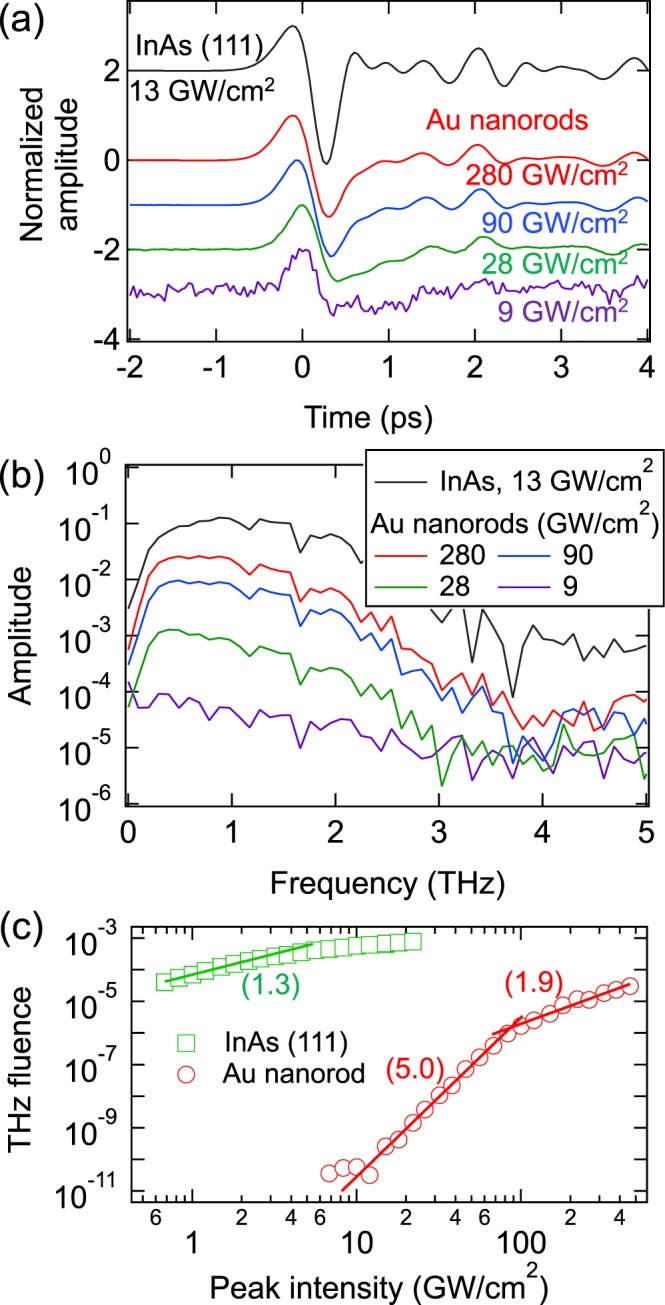
Reflection geometry, excitation at 800 nm. (**a**) Time-domain waveforms and (**b**) corresponding amplitude spectra for different peak optical intensities. Each waveform is normalized to its maximum positive value. (**c**) Terahertz pulse energy as a function of the peak optical intensity. The curves for the InAs (111) surface are shown for comparison. In (**c**), the numbers indicate the slopes of the fitted lines in a log scale.

In the reflection geometry, the base Au film makes the Au nanorod sample opaque for terahertz waves; therefore, they are mostly radiated in the reflection direction. The measured time-domain waveforms, corresponding Fourier transformed amplitude spectra, and optical intensity dependence of the terahertz pulse energy are shown in Fig. 3 in comparison with those from one of the best surface emitters, i.e., a surface of InAs (111)^17^. The time-domain waveforms (Fig. 3(a)) are very similar for both emitters, despite the differences in the terahertz generation mechanisms. According to our experimentally measured azimuthal dependence of the terahertz radiation amplitude, terahertz emission from InAs can be mainly attributed to surface electric-field-induced optical rectification^18,19^, rather than photoejected electrons. The waveform we obtained from InAs (Fig. 3(a)) agrees well with that in ref.^19^. In Fig. 3(b), the spectral bandwidth is limited by about 3 THz because of the optical-terahertz phase mismatch in the 1-mm thick ZnTe (110) detector crystal^20^.

According to Fig. 3(c), terahertz emission from the Au nanorod sample was observed when the peak optical intensity was above ~10 GW/cm^2^. For the intensities between ~10 and ~100 GW/cm^2^, the terahertz pulse energy exhibits a 5th order dependence on the peak optical intensity. For the intensities above ~100 GW/cm^2^, the order of the dependence reduces to 1.9. For these high intensities, terahertz emission from the Au nanorod sample becomes comparable to that from the InAs surface observed at much lower intensities ~1 GW/cm^2^. Terahertz emission from InAs is already saturated at ~1 GW/cm^2^ ^21^, and exhibits a 1.3 order dependence on the peak optical intensity.

Similar bimodal intensity dependences, with a reduction of the nonlinear order from ~3–6 to ~1.5–2, have also been observed in some previous papers^2,3,6,9^. The higher order (~3–6) nonlinearity has been attributed to the multiphoton ionization and ponderomotive acceleration of photoejected electrons by plasmonically enhanced optical near field^3,6,9,14^. In this model, the generated terahertz electric field *E*_*THz*_ can be expressed as *E*_*THz*_ ∝ *N*(*I*_*ex*_) × *a*(*I*_*ex*_), where $N({I}_{ex})\propto {I}_{ex}^{n}$ is the photoelectron number with *n* the multiphoton ionization order, *a*(*I*_*ex*_) ∝ *I*_*ex*_ is the photoelectron acceleration, and *I*_*ex*_ is the optical excitation intensity. The multiphoton ionization order *n* can be found through the metal work function *W* and the photon energy *ħω* as *n* ∼ *W*/(*ħω*). Thus, for the excitation of gold (*W* ∼ 5.1 eV) with a Ti:sapphire laser at 800 nm (*ħω* ∼ 1.55 eV) one can expect that *n *~ 3 and the terahertz intensity scales as ${I}_{THz}\propto {|{E}_{THz}|}^{2}\propto {I}_{ex}^{8}$. This prediction does not agree with Fig. 3(c) and previous experimental results^3,6^. To explain the inconsistency, it was proposed that plasmonic enhancement of the optical near-field is strong enough to provide a transition between multiphoton to tunneling regimes of electron emission and, therefore, a reduction of the overall nonlinear order of the terahertz generation process^6,14^.

**Figure 4:**
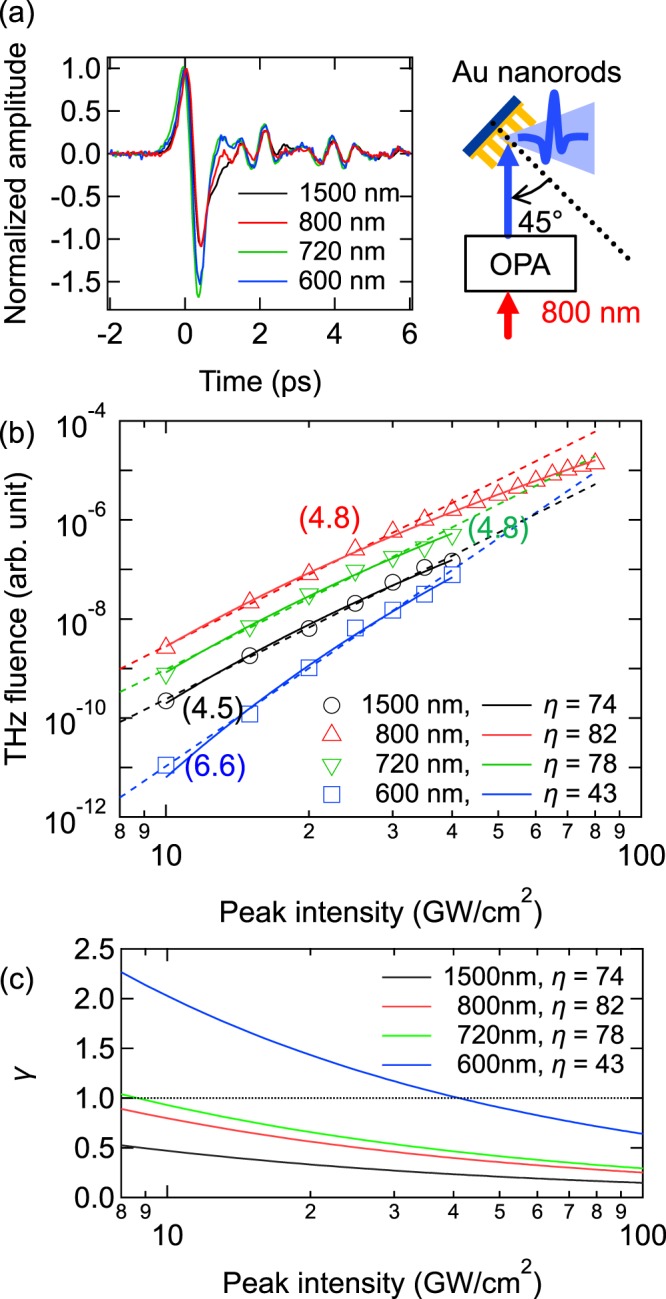
Reflection geometry, excitation at different wavelengths. (**a**) Time-domain waveforms for the peak optical intensity of 40 GW/cm^2^. (**b**) Terahertz pulse energy as a function of the peak optical intensity. The dashed lines and numbers near them indicate the power laws and their orders, respectively. The solid lines are fitting with Eq. (1). Values of the enhancement factor *η* are shown in legend. (**c**) Parameter *γ* as a function of the peak optical intensity. Inset: schematic of the experiment.

To verify this explanation, we made an experiment on terahertz emission from the Au nanorod sample under excitation at different wavelengths. Schematic of the experiment is shown in inset in Fig. 4(a). An optical parametric amplifier (OPA) was used as a pump. The sample was illuminated from the air side (reflection geometry) by a *p*-polarized light at a fixed incident angle of 45°.

Figure 4(a) shows the measured time-domain waveforms for several excitation wavelengths: 600, 720, 800, and 1500 nm. The peak optical intensity was estimated as 40 GW/cm^2^ under the assumption that the beam diameter and pulse duration of the OPA output radiation remain the same as those of the 800-nm exciting laser. According to Fig. 4(a) the waveforms are very similar for all the excitation wavelengths.

Figure 4(b) shows the terahertz pulse energy as a function of the peak optical intensity. Unexpectedly, although the wavelengths 600, 720, 800, and 1500 nm correspond to considerably different photon energies, i.e., 2.01, 1.72, 1.55, and 0.83 eV, respectively, the nonlinear order of the generation process varies insignificantly with the wavelength. Moreover, the order is even somewhat higher for the shorter wavelength of 600 nm.

## Discussion

To explain the observed weak dependence of the nonlinear order of the generation process on the excitation wavelength, we invoke the transition between multiphoton to tunneling regimes of electron emission. This transition is characterized by the Keldysh parameter^22^, $\gamma =\omega \sqrt{2{m}_{e}W}/(e{E}_{s})$, where *m*_*e*_ and *e* are the electron mass and charge, respectively, and *E*_*s*_ is the electric field on the metal surface. The electric field of the excitation laser *E*_*ex*_ can be evaluated from the laser intensity by using formula ${E}_{ex}=\sqrt{2{I}_{ex}/(c{\epsilon }_{0})}$, where *c* is the speed of light and ${{\epsilon }}_{0}$ is the vacuum permittivity. If one takes *E*_*s*_ ~ *E*_*ex*_ to estimate the parameter γ, the intensity interval *I*_*ex*_ ~ 10–100 GW/cm^2^ at the wavelength of ~800 nm corresponds to *γ *~ 70–20, i.e., rather to multiphoton ($\gamma \gg 1$) than tunneling (*γ* < 1) regime of electron emission. In Fig. 4(b), the nonlinear order is, however, almost independent of the wavelength. This indicates unambiguously that tunneling dominates over multiphoton emission, i.e., *γ* < 1. It can be explained by taking into account a plasmonic enhancement of the electric field in the vicinity of the nanorods. The enhancement increases the field *E*_*s*_ = *ηE*_*ex*_ by a factor of $\eta \gg 1$, thereby reducing the Keldysh parameter *γ*.

In the tunneling regime (*γ* < 1), the electron emission from a metal surface adiabatically follows the instantaneous electric field *E*_*s*_. The emission current density can be described by the Fowler-Nordheim equation^23,24^

1 $$J({E}_{s})=\frac{{e}^{3}{E}_{s}^{2}}{16{\pi }^{2}\hslash W}\exp (-\frac{4}{3}\frac{\sqrt{2{m}_{e}}{W}^{3/2}}{\hslash e{E}_{s}}),$$

which is independent of the optical pump wavelength. Assuming further that the terahertz intensity *I*_*THz*_ scales as *I*_*THz*_ ∝ *J* ^2^, we fit the experimental dependences in Fig. 4(b) with Eq. (1) using *η* as a fitting parameter. The fitting reproduces well not only the power laws of the dependences but also the saturation, which is observed at ~100 GW/cm^2^ for the 800 nm excitation.

The fitted enhancement factor *η* is almost the same (*η* ∼ 70−80) for 720, 800, and 1500 nm, but considerably smaller (*η* ∼ 40) for 600 nm. This agrees well with the resonance curve in Fig. 1(c), where the plasmon resonance peaks at 800 nm and spreads to the longer wavelengths, while the wavelength of 600 nm is out of resonance. The field enhancement factors as high as several dozens have been already reported even for regular arrays of gold nanorods^12,25–27^. In a nanoforest structure with randomly inclined nanorods, such as our sample (Fig. 1(b)), there exist hot spots induced by plasmonic coupling between nanorods, which can provide much higher enhancement factors^28–30^. Taking also into account that the work function of Au can be smaller depending on the sample morphology^31^, the estimated order of the enhancement factor seems reasonable.

Figure 4(c) shows the intensity dependences of the Keldysh parameter *γ* with the plasmonic enhancement taken into account, i.e., with *E*_*s*_ = *ηE*_*ex*_ and the fitted values of *η*, for different excitation wavelengths. For the wavelengths of 720, 800, and 1500 nm, parameter *γ* is smaller than 1 in the whole intensity range. For 600 nm, *γ* is around 1. This confirms that the tunneling mechanism of the electron emission dominates over the multiphoton mechanism and thereby explains the weak dependence of the nonlinear order on the wavelength.

From the fitting of the experimental results, one can make following conclusions. First, high-order (~5–6th) dependences of the terahertz fluence on the optical intensity are not inevitably related to the multiphoton regime of electron photoemission. Such dependences can be observed in the tunneling regime (in the excitation intensity range of 10–100 GW/cm^2^) due to the exponential factor in the Fowler-Nordheim Eq. (1). In particular, the higher (~6.6) order of the dependence for 600 nm can be explained by lower values of *E*_*s*_ (due to a smaller plasmonic enhancement at 600 nm) and, therefore, stronger influence of the exponential factor in Eq. (1). Second, the model of direct terahertz emission from the tunneling current (1), with reasonable values of the enhancement factor and without invoking a ponderomotive acceleration of the ejected electrons, reproduces well the experimental results. Our attempts to include the ponderomotive mechanism by introducing an additional factor ${I}_{ex}^{2}$ to the formula for the generated terahertz intensity, i.e., ${I}_{THz}\propto {I}_{ex}^{2}{J}^{2}$, led to unrealistically high (of several hundreds) values of the enhancement factor *η*, when the formula was used for fitting the experimental results. Thus, our results are in favor of the following model. Tunneling ionization and direct acceleration of electrons by plasmonically enhanced individual half-cycles of the sinusoidal optical field produce sub-cycle current bursts *J*(*t*) in the vicinity of the metal. The bursts follow adiabatically the instantaneous optical field according to Eq. (1) and occur mainly near the peak intensity of the laser pulse. As a result, an individual laser pulse produces a surge of an average current $\langle J\rangle ={\tau }^{-1}{\int }_{0}^{\tau }J(t)dt$ on a time scale shorter than the laser pulse duration *τ*. This current surge acts as an emitter of terahertz radiation. The current surge generates very broadband terahertz radiation, which, however, cannot be detected in the whole spectrum by means of the standard electro-optic sampling with a ZnTe crystal. This conception agrees with the experimental observations of sub-cycle regimes of photoemission from gold nanotips^32^ and nanoparticles^27^.

## Conclusion

We measured terahertz emission from an array of gold nanorods illuminated by intense (~10–100 GW/cm^2^) femtosecond optical pulses of different central wavelengths. We found, for the first time, that the order of the dependence of the terahertz fluence on the optical intensity varies only slightly with changing the wavelength in a wide range: The order is almost the same (~4.5–4.8) for 720, 800, and 1500 nm and somewhat higher (~6.6) for 600 nm. These observations unambiguously demonstrate that tunneling regime of photoemission dominates over multiphoton regime. The dependences of the terahertz fluence on the optical intensity are fitted well under the assumption that terahertz emission originates directly from the tunneling current given by the Fowler-Nordheim equation with reasonable values of the plasmonic enhancement factor. Fitting with additionally included ponderomotive acceleration of the photoejected electrons requires unrealistically high values of the enhancement factor. Based on these findings, we suggest that terahertz radiation originates from the sub-cycle bursts of the tunneling current rather than the ponderomotively accelerated electrons.

## Data Availability

All data generated or analyzed during this study are included in this article.
